# Migraine without aura: genome-wide association analysis identifies several novel susceptibility

**DOI:** 10.1186/1129-2377-14-S1-P21

**Published:** 2013-02-21

**Authors:** B Loci De Vries, T Freilinger, V Anttila, R Malik, GM Terwindt, P Pozo-Rosich, B Winsvold, D Nyholt, WPJ van Oosterhout, V Artto, M Todt, E Hämäläinen, J Fernandez-Moralez, M Louter, MA Kaunisto, J Schoenen, O Raitakari, T Lehtimäki, M Ville-Pueyo, H Göbel, E Wichman, C Sintas, A Uitterlinden, A Hofman, F Rivadeneira, A Heinze, E Tronvik, CM van Duin, J Kaprio, B Cormand, M Wessman, RR Frants, T Meitinger, B Müller-Myhsok, JA Zwart, M Färkkilä, A Macaya, MD Ferrari, C Kubisch, A Palotie, M Dichgans, AMJ van den Maagdenberg

**Affiliations:** 1 Department of Human Genetics, Leiden University Medical Centre (LUMC), Netherlands; 2 Institute for Stroke and Dementia Research, Klinikum der Universität München, Munich, Germany; 3 Wellcome Trust Sanger Institute, Wellcome Trust Genome Campus, Cambridge, UK; 4 Department of Neurology, Leiden University Medical Centre (LUMC), Netherlands; 5 Department of Neurology, Vall d'Hebron University Hospital, Universitat Autònoma de Barcelona, Spain; 6 Department of Neurology, Oslo University Hospital and University of Oslo, Norway; 7 Neurogenetics Laboratory, Queensland Institute of Medical Research, Brisbane, Australia; 8 Department of Neurology, Leiden Medical Centre (LUMC), Netherlands; 9 Department of Neurology, Helsinki University Central Hospital, Helsinki, Finland; 10 Institute of Human Genetics, University of Ulm, Germany; 11 Institute for Molecular Medicine Finland (FIMM), University of Helsinki, UK; 12 Headache Research Unit, Department of Neurology and Groupe Interdisciplinaire de Génoprotéomique Appliquée (GIGA)-Neurosciences, Belgium; 13 Department of Clinical Physiology, University of Turku and Turku University Central Hospital, Turku, Finland; 14 Department of Clinical Chemistry, Tampere University Hospital and University of Tampere, Tampere, Finland; 15 Pediatric Neurology Research Group, Vall d'Hebron Research Institute, Barcelona, Spain; 16 Kiel Pain and Headache Center, Kiel, Germany; 17 Institute of Epidemiology, Helmholtz Center Munich, Neuherberg, Germany; 18 Department of Genetics, University of Barcelona, Barcelona, Spain; 19 Department of Internal Medicine, Erasmus Medical Center, Rotterdam, Netherlands; 20 Department of Epidemiology, Erasmus University Medical Center, Rotterdam, Netherlands; 21 Department of Neuroscience, Norwegian University of Science and Technology, Trondheim, Norway; 22 Institute for Molecular Medicine Finland (FIMM), University of Helsinki, Finland; 23 Institute of Human Genetics, Helmholtz Zentrum München, Neuherberg, Germany; 24 Max Planck Institute of Psychiatry, Munich, Germany; 25 Oslo University Hospital and University of Oslo, Norway

## Introduction

Genome-wide association studies (GWAS) are a novel and promising method to study genetic susceptibility factors for common disorders, including migraine.

## Objective

Here we performed the first GWAS in migraine without aura (MO), which is the most common form of migraine.

## Methods

To identify common genetic variants for this migraine type, we analyzed genome-wide association data of 2,326 clinic-based German and Dutch patients and 4,580 population-matched controls. Loci with two or more SNPs with P-values < 1 x 10-5 were selected for follow-up in 2,508 Dutch, Spanish, Finnish and Norwegian patients and 2,652 controls.

## Results

Meta-analysis of the discovery and replication data yielded four genome-wide significant (P < 5 x 10-8) MO susceptibility loci in or nearby MEF2D, PHACTR1, ASTN2 and TGFBR2. In addition, SNPs in two loci (in or near TRPM8 and LRP1) that were previously identified in a GWAS on population-based migraine were significantly replicated in our clinic-based MO cohort.

## Conclusion

This study reveals the first susceptibility loci for migraine without aura, thereby expanding our knowledge of this debilitating neurological disorder.

